# Acute bilateral myopia induced by Triplixam: a case report

**DOI:** 10.1186/s12886-020-01635-2

**Published:** 2020-09-22

**Authors:** Ruta Jaruseviciene, Ginte Sirvydyte

**Affiliations:** Center of Eye Diseases, Clinic of Ear, Nose, Throat and Eye Diseases, Institute of Clinical Medicine, Faculty of Medicine, Vilnius University, Vilnius, Lithuania

**Keywords:** Myopia, Sulphonamide, Triplixam, Transient, Sudden

## Abstract

**Background:**

Side effects of the systemic drugs used to treat eyes are not common. Triplixam is used to treat systemic hypertension and contains amlodipine, indapamide and perindopril arginine as active ingredients which might have induced the sudden myopia. The transient myopia with objective findings disappeared after the discontinuation of the drug.

**Case presentation:**

A 33-year-old male presented to the emergency department with a history of blurred vision in both eyes. Development of myopia, lens thickening, choroidal effusion and retinal striae at the macula with the increase in macular thickness was observed in both eyes. These symptoms cleared completely after the drug was discontinued. Myopisation could have been caused by lens thickening and changing its refractive index as a result of allergic or idiosyncratic reaction of the ciliary body. Retinal striae may be caused by the volume effect of the choroidal effusion.

**Conclusion:**

Our report describes the adverse effect of Triplixam, probably resulting from its ingredient indapamide. Although indapamide is a common drug used in the treatment of systemic hypertension, it is important for cardiologists, general practitioners and other physicians to be aware of the possible adverse effect of Triplixam.

## Background

Ocular side-effects induced by drugs are rare. Transient myopia induced by drugs has been reported to be caused by the ingestion of sulphonamide-derived drugs, such as methazolamide, sulfasalazine, indapamide, acetazolamide, hydrochlorothiazide, ethoxzolamide, psychotropic drugs, etc. [1]. In this paper a case of sudden vision blurring in a young male who used Triplixam is presented. Triplixam is a drug used to treat systemic hypertension that combines three active ingredients: amlodipine, indapamide and perindopril arginine. Indapamide is a sulphonamide-derived diuretic which might have induced the myopia. The transient myopia disappeared after the discontinuation of the drug.

## Case presentation

**Fig. 1:**
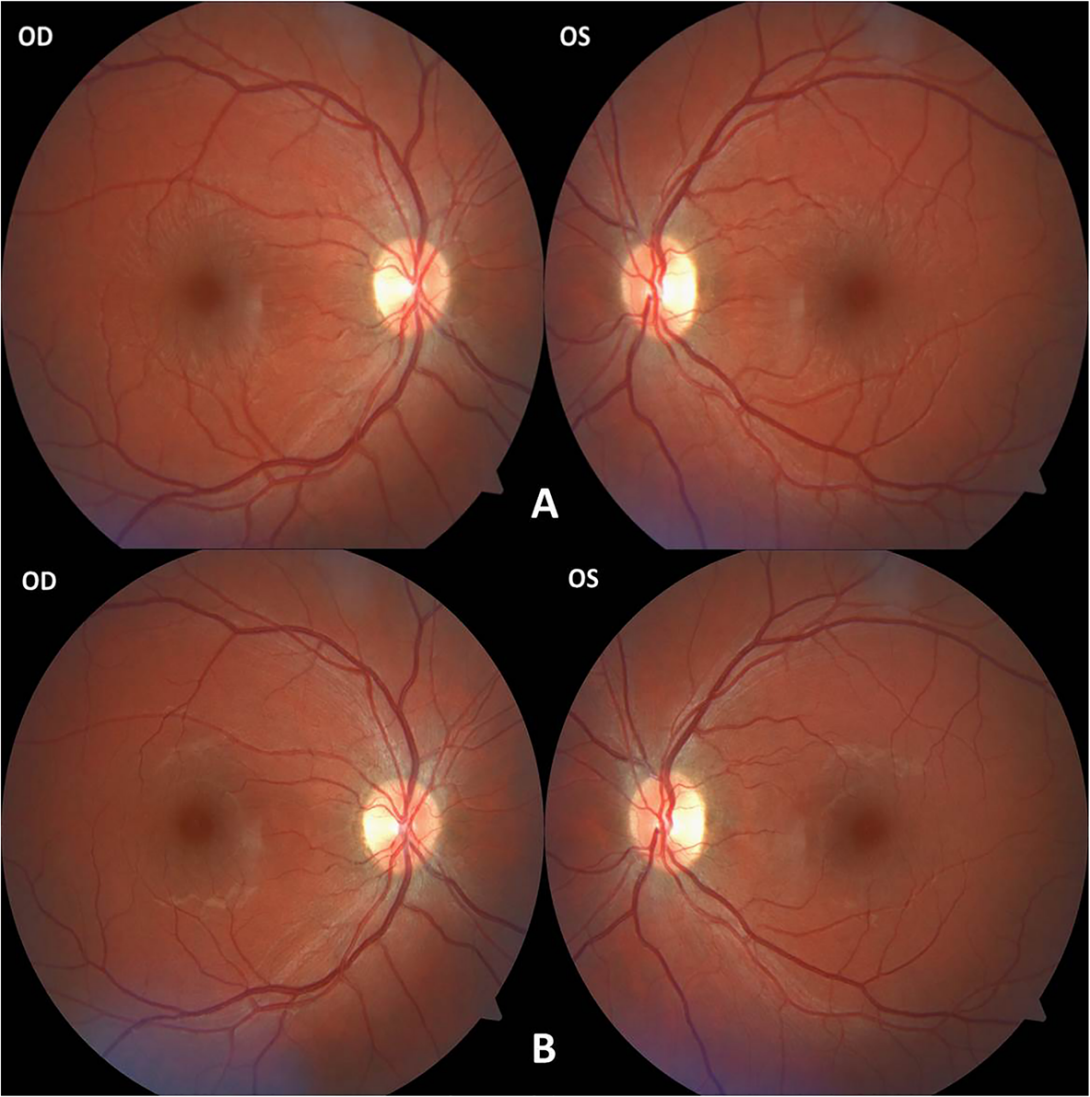
Retinal striae radiated from the fovea in the both eyes on the first visit (**a**) and disappearance on control visit (**b**) in fundus photography

**Fig. 2:**
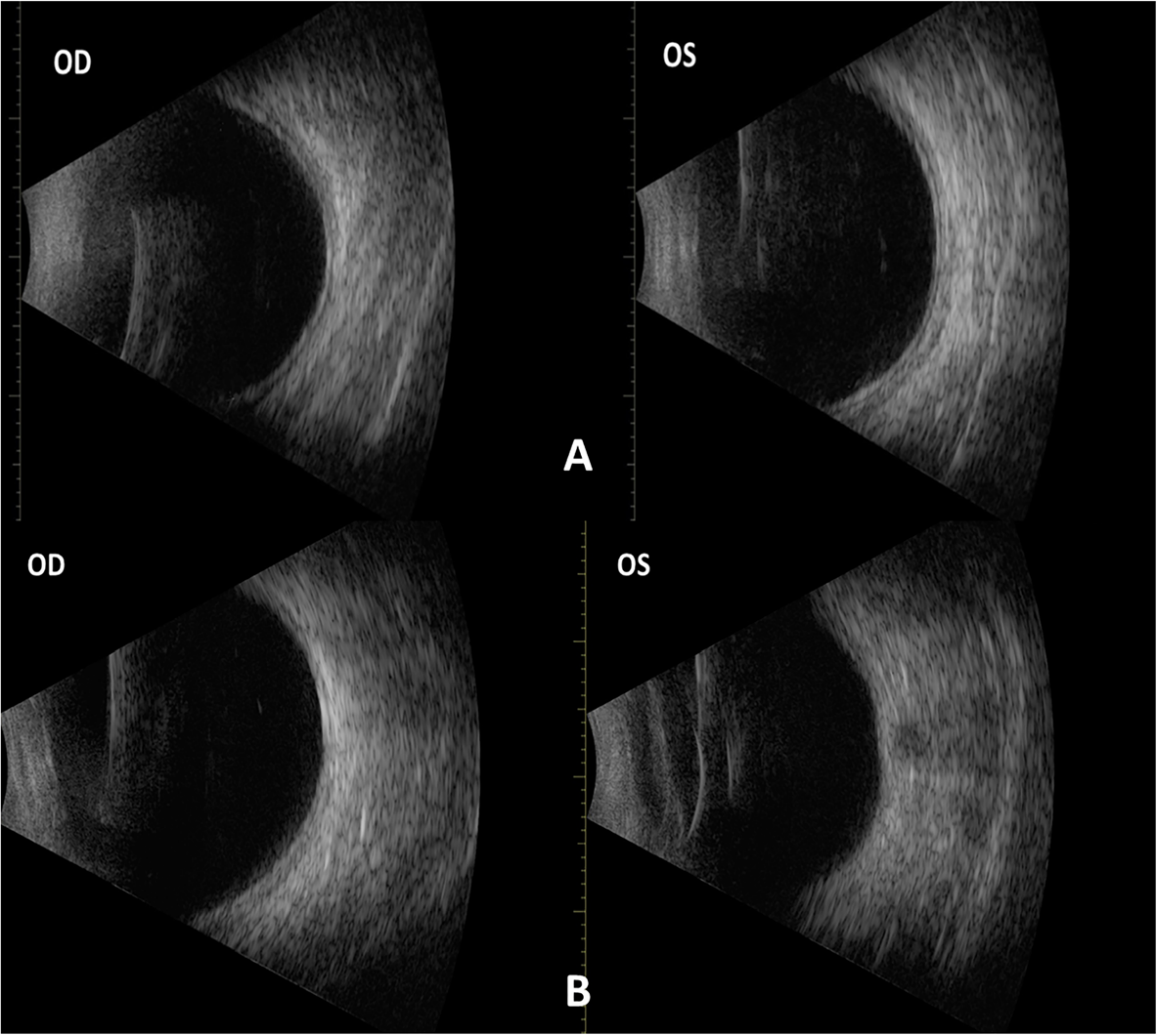
Diffuse choroidal thickening in the both eyes on the first visit (**a**) and disappearance on control visit (**b**) in B scan ultrasonography

**Fig. 3:**
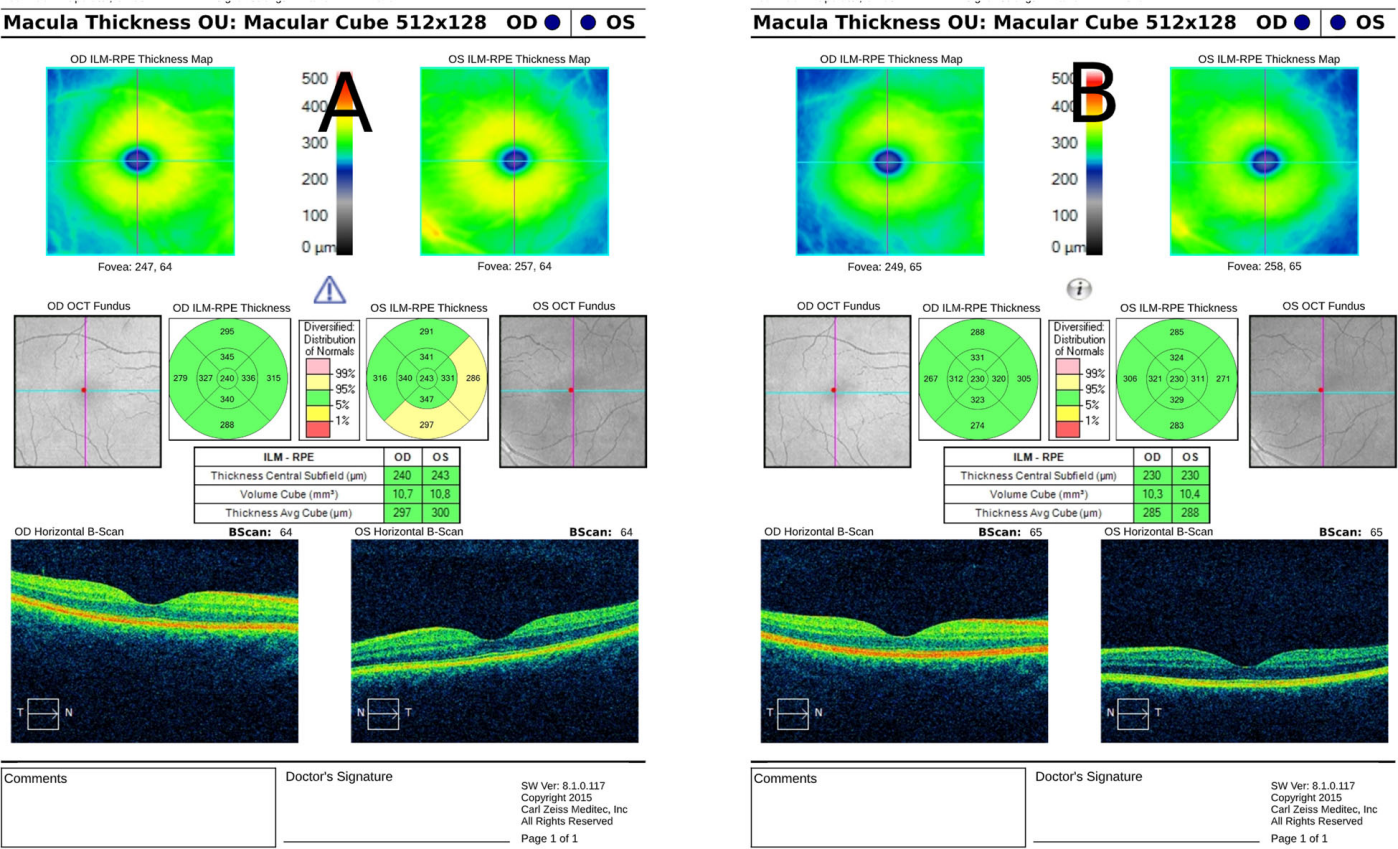
Thickening of the macula in the both eyes on the first visit (**a**) and reduction on control visit (**b**) in OCT scan

A 33-year-old male presented to the emergency department with a history of blurred vision in both eyes. The patient started experiencing blurred vision in the morning after using a computer during the night before. The patient did not feel any pain or other symptoms and the patient had never worn glasses. The medical history of the patient revealed uncontrolled hypertension that had lasted for several years for which he had started taking medications recently. He was taking Triplixam (amlodipine 5 mg, indapamide 2.5 mg, perindopril arginine 5 mg) (Les Laboratoires Servier, France) once per day, prescribed by his physician for his systemic hypertension. He started this medication 4 days prior to the appearance of vision blurring. The visual acuity at presentation was 20/40 in the right eye and 20/100 in the left eye. The intraocular pressure was 14.6 mmHg in both eyes (determined by using a Schiøtz indentation tonometer). The results of slit lamp biomicroscopy showed that the cornea, lens, and vitreous were normal, however, a retinal striae radiated from the fovea in the both eyes. Fundus photography was performed to document the retinal striae (Fig. 1a). After performing a refraction test, the visual acuity of the patient improved to 20/20 in both eyes with a spherical correction of − 1.75 dioptre sphere (DS) in the right eye and − 2.00 DS in the left eye. Objective refraction was − 1.75 DS/− 0.50 dioptre cylinder (DC) × 21° in the right eye and − 2.00 DS/− 0.25 DC × 165° in the left eye. A cycloplegic refraction revealed a value of − 1.50 DS/− 0.25 DC × 36° in the right eye and − 2.00 DS/− 0.25 DC × 136° in the left eye. The central corneal thickness and corneal endothelial morphology were evaluated using non-contact specular microscopy and there were no pathological changes observed. The initial B-scan ultrasonography showed diffuse choroidal thickening (Fig. 2a). Optical biometry was performed to measure the axial length which was 23.70 mm and 23.76 mm in the right and the left eye, respectively. The lens thickness was 4.19 mm and 4.18 mm in the right and the left eye, respectively. The anterior chamber depth was similar in both eyes at 3.60 mm. An optical coherence tomography (OCT) showed a mild thickening of the macula at 240 and 243 μm in the right and the left eye, respectively (Fig. 3a).

The patient was advised to stop taking a triple drug combination, Triplixam, and was referred to a cardiologist for a further investigation. The patient reported that blurred vision reversed 4 days after discontinuing the medication. After 16 days, the patient was examined once again as he was travelling abroad due to a planned vacation. His visual acuity had improved to 20/20 without any refractive error. Intraocular pressure was 15.9 mmHg in both eyes (determined by using a Schiøtz indentation tonometer). Refraction results revealed a value of 0.00 DS/− 0.25 DC × 15° in the right eye and 0.00 DS/− 0.25 DC × 165° in the left eye. Repeated measurements of endothelial morphology and corneal thickness showed no significant differences. Optical biometry revealed no difference in axial length. The lens thickness decreased to 3.97 mm and 3.96 mm in the right and the left eye, respectively. The anterior chamber depth increased to 3.68 mm and 3.69 mm in the right and the left eye, respectively. Fundus examination revealed the disappearance of retinal striae at the macula and peripheral choroidal effusion had also resolved in B-scan. (Figs. 1b and 2b). The OCT was repeated which showed a reduction in the thickening of the macula, 230 μm in both eyes (Fig. 3b). A presumptive diagnosis of drug-induced acute myopia due to an ingredient of Triplixam – diuretic indapamide – was made.

Electrocardiography, veloergometry and echocardiography tests were performed, and no pathology was revealed. Patient was prescribed by a cardiologist to take 1 mg of Rilmenidine per day to control arterial hypertension.

## Discussion and conclusions

We report a case of acute myopia caused by oral consumption of Triplixam which is a combination of three active ingredients: amlodipine, indapamide and perindopril arginine. Triplixam is used to treat systemic hypertension. There are reports that diuretics can be the cause of acute myopia; the active ingredient indapamide was the most likely cause of acute myopisation [2, 3]. Other authors have reported a case of an acute myopia when patient was treated for arterial hypertension with a combination of indapamide and amlodipine and all symptoms reversed when only amlodipine was left to control hypertension [4]. These findings exclude the possible myopic effect of amlodipine. Also, there is no evidence that perindopril arginine could cause acute myopia. Indapamide is a sulphonamide diuretic and is generally used to treat hypertension. The mechanism of myopisation is not completely understood. Literature describes paradoxical cases of idiopathic oedema caused by diuretic therapy [5]. Myopisation might have been caused by a lens thickening and changing its refractive index as a result of an allergic or idiosyncratic reaction of the ciliary body [3, 6, 7]. Previously mentioned authors revealed that a more precise ultrasound biomicroscopy and mode B ultrasonography disclosed bilateral ciliochoroidal effusion with anterior rotation of the ciliary body and iridocorneal angle narrowing, possibly resulting in sudden myopia [4]. The reported retinal striae may have been caused by vitreoretinal traction [8]. Other authors report the presence of striae due to internal limiting membrane folds caused by the volume effect of the choroidal effusion [9]. In the analysed case, cycloplegics had no effect and the diagnosis of myopia in association with ciliary spasm was withdrawn. The patient did not report severe eye pain, his intraocular pressure was within the normal range and no other acute angle-closure glaucoma symptoms were observed. After discontinuing Triplixam, we believe that no further treatment is required. Patient was referred to a cardiologist to change the drug group in order to control arterial hypertension.

Indapamide in combination with other ingredients of Triplixam was probably the sole cause of this adverse reaction. Although indapamide is a common drug used to treat systemic hypertension, it is important that cardiologists, general practitioners and other physicians are aware of possible adverse effects of Triplixam.

## Data Availability

The datasets analysed during the current study are available from the corresponding author on reasonable request.

## Abbreviations

DS: Dioptre sphere
DC: Dioptre cylinder
OCT: Optical coherence tomography
